# Near Real-Time Wildfire Progression Monitoring with Sentinel-1 SAR Time Series and Deep Learning

**DOI:** 10.1038/s41598-019-56967-x

**Published:** 2020-01-28

**Authors:** Yifang Ban, Puzhao Zhang, Andrea Nascetti, Alexandre R. Bevington, Michael A. Wulder

**Affiliations:** 1 Division of Geoinformatics, KTH Royal Institute of Technology, 10044 Stockholm, Sweden; 2 British Columbia Ministry of Forests, Lands, Natural Resource Operations and Rural Development, Prince George, British Columbia V2M 2H3 Canada; 3 Canadian Forest Service (Pacific Forestry Centre), Natural Resources Canada, Victoria, British Columbia V8Z 1M5 Canada

**Keywords:** Natural hazards, Computational science

## Abstract

In recent years, the world witnessed many devastating wildfires that resulted in destructive human and environmental impacts across the globe. Emergency response and rapid response for mitigation calls for effective approaches for near real-time wildfire monitoring. Capable of penetrating clouds and smoke, and imaging day and night, Synthetic Aperture Radar (SAR) can play a critical role in wildfire monitoring. In this communication, we investigated and demonstrated the potential of Sentinel-1 SAR time series with a deep learning framework for near real-time wildfire progression monitoring. The deep learning framework, based on a Convolutional Neural Network (CNN), is developed to detect burnt areas automatically using every new SAR image acquired during the wildfires and by exploiting all available pre-fire SAR time series to characterize the temporal backscatter variations. The results show that Sentinel-1 SAR backscatter can detect wildfires and capture their temporal progression as demonstrated for three large and impactful wildfires: the 2017 Elephant Hill Fire in British Columbia, Canada, the 2018 Camp Fire in California, USA, and the 2019 Chuckegg Creek Fire in northern Alberta, Canada. Compared to the traditional log-ratio operator, CNN-based deep learning framework can better distinguish burnt areas with higher accuracy. These findings demonstrate that spaceborne SAR time series with deep learning can play a significant role for near real-time wildfire monitoring when the data becomes available at daily and hourly intervals with the launches of RADARSAT Constellation Missions in 2019, and SAR CubeSat constellations.

## Introduction

Research shows that human-induced climate changes have led to hot, dry conditions that increase the outbreaks of wildfires in various regions^1^. In both 2017 and 2018, the world witnessed many devastating wildfires. Hotter summers and drought across northern Europe and North America have resulted in increased wildfire activity in cooler and wetter regions such as Sweden, even north of the Arctic Circle. During the summer of 2018, Sweden experienced an exceptionally long period of drought resulting in over 50 forest fires. In British Columbia, Canada, a total of 2,092 wildfires burnt more than 1.3 million hectares of land in 2018, while in California, USA, a total of 8,527 wildfires burned an area of 800 000 hectares, the deadliest and most destructive wildfire season in California’s history. Early identification of the location and size of these fires can inform fire-fighting operations at an early stage. Moreover, being able to monitor wildfire progression and burnt areas in both cloudy and smoky conditions as well as during day and night would increase the information base for decision-making and improve the speed and efficiency for wildfire emergency response. With its synoptic view and large area coverage at regular revisits, satellite remote sensing has long played a crucial role in disaster management including wildfire. Owing to the rapid development of satellite technology, we are moving forward to a new era of Earth Observation (EO). National and International space agencies, as well as innovative companies have started various EO programs that are able to acquire massive amounts of satellite imagery with increasingly higher spatial resolution and rapid temporal intervals. With the recent launches of the European Space Agency (ESA)’s Sentinel-1 and Sentinel-2 satellites, SAR and optical data with global coverage and frequent revisits have become freely available. These open EO big data represent a great opportunity to develop innovative methodologies for near real-time wildfire monitoring. The main challenge is the lack of robust and automated methods to extract relevant information from such massive amounts of EO data.

For active wildfire monitoring, the low spatial resolution Visible Infrared Imaging Radiometer Suite (VIIRS) and the Moderate Resolution Imaging Spectroradiometer (MODIS) are often used for preliminary mapping and contextual awareness while Landsat and Sentinel-2 data are deployed for post-fire boundary determination and burn severity mapping^2–5^. Optical images at critical time frame, however, are often unavailable due to frequent cloud cover in the boreal and tropical regions^6^. As an active sensing technology, not relying on solar radiation, SAR is capable of penetrating clouds and smoke as well as imaging day and night^7^. As a result, SAR has been playing an increasingly important role in environmental change monitoring. Past studies evaluated various SAR data at X-, C-, and L-bands in different polarizations for burnt area mapping and burn severity estimation^8–17^. For examples, Bourgeau-Chavez *et al*.^17^ evaluated European remote sensing satellite (ERS) C-band SAR for detection of boreal fire scars in various boreal ecosystems globally was possible using C-band SAR data. Gimeno *et al*., identified burnt areas in Mediterranean forests using Principle Component Analysis (PCA) and neural network classification^18^. Polychronki *et al*. evaluated Advanced Land Observing Satellite (ALOS) the Phased Array type L-band Synthetic Aperture Radar (PALSAR) imagery for burnt area mapping using an object-based classification in Greece^13^. Tanase *et al*. investigated spaceborne X-, C- and L-band co- and cross-polarized data for burn severity estimation in Spanish pine forests^16^. Previous results reported a substantial decrease of the C-band VH backscatter in fire-disturbed forests and demonstrated the effectiveness of cross-polarized SAR for detection of burn scars^11,19^. Conversely, several C-band SAR-based studies revealed an increase in co-polarized backscatter from fire-disturbed forests in boreal and Mediterranean due to rain-fall events and low evapotranspiration^10,13–15,17,20,21^. As a result of the partial or complete removal of forest canopy, changes in the soil moisture have a significant effect on SAR backscatter. C-band SAR backscatter from fire scars were observed to 3–6 dB higher than the adjacent unburnt forest in Alaska when the soil was wet. Similar backscatter behaviors caused by soil moisture changes were also observed in fire-disturbed forests in various environmental conditions around the world, including Australia, Canada, Indonesia, the Mediterranean and Siberia^17,18,20,22,23^.

The capability of Sentinel-1 for fire scar mapping was investigated in recent studies with mixed results^8,11,24^. According to Imperatore *et al*.^11^, there is an obvious change in VH backscatter for the burnt area dominated by forests, thus, permitting the delineation of the fire-affected areas. However, the detection of the burnt areas for grass and non-forest vegetation is more complex. C-band SAR backscatter of burnt grass is similar to that of dry grass. The main challenges of using SAR for detecting burnt areas and mapping fire scars are related to the complex interactions of SAR system parameters, including frequency, polarization, incidence angles and look direction, and land surface parameters, including geometric properties (type and structure, surface roughness, geometry and topography etc.) and dielectric properties (i.e., surface moisture), as well as environmental conditions (e.g., rain, dew, wind etc.). Therefore, further research is needed to better understand the temporal backscatter behavior of Sentinel-1 C-Band SAR in VV and VH polarizations for burnt forest and grassland compared to that of burnt areas. Spaceborne SAR data and their fusion with Landsat data were also investigated for near-real time deforestation monitoring^25–27^. To the best of our knowledge, however, no study was found on SAR for wildfire progression monitoring. Therefore, it is desirable to investigate Sentinel-1 SAR dense time series data for wildfire progression monitoring. Wildfires burn vegetation thus result in changes in the SAR backscatter intensity that can be identified with change detection.

Change detection has been used successfully in many diverse applications, including monitoring environmental changes, land-use/land-cover dynamics, deforestation, disaster damage, and urbanization^28–33^. Various change detection techniques have been developed, such as those by Bruzzone and Prieto^34^, Bazi *et al*.^35^, Bovolo and Bruzzone^36^ and Bovolo *et al*.^37^. However, most of these methods and algorithms were developed considering the availability of a few or just two images (pre- and post-event) that can be used to detect the changes. Change detection remains a challenging task using SAR data due to complexity of wildfire affected forest environment. Some of the common issues of change detection, however, could be resolved by exploiting the high temporal resolution of Sentinel-1 time series using the highly redundant information following the big data paradigm. The idea is to exploit the temporal fluctuations of the remotely sensed measurements to remove the confusion between burnt areas and other changes. As one of the fastest-growing trends in big data analytics, deep learning is proving to be a very effective technique for large-scale image recognition^38–40^ and semantic segmentation^41^. Among deep learning models, the most exciting is the potential of CNN in learning complex non-linear transformation and extracting mid- and high-level feature representations from raw image pixels by interleaving convolutional and pooling layers. Recent studies highlight the potential to extract changes from EO data using deep neural networks^42–45^. Therefore, CNN-based framework and other deep learning methods are investigated in this research.

In this research, we investigate Sentinel-1 SAR dense time series for near real-time wildfire progression monitoring through smoke, cloud and night using a deep learning-based framework. Our specific objectives are: (1) better understand the temporal backscatter patterns of burnt forest and grassland; (2) evaluate Sentinel-1 SAR dense time series for near real-time wildfire progression monitoring; (3) develop a CNN-based deep learning framework that could efficiently and automatically detect burnt areas using the SAR dense time series data; and (4) validate the proposed deep learning framework in three different study areas, the Elephant Hill Fire (2017) in British Columbia of Canada, the Camp Fire (2018) in California, U.S.A., and the Chuckegg Creek Fire (2019) in northern Alberta of Canada.

## Study Areas and Data Description

### Study areas

Three recent wildfires, the Elephant Hill wildfire (Canada, 2017), the Camp Fire (U.S.A., 2018) and the Chuckegg Creek Fire (Canada, 2019), were selected for this research to represent different environmental conditions. The Elephant Hill wildfire (Canada) was British Columbia’s largest wildfire in 2017. Started on July 6 along the Thompson River near Ashcroft and contained in mid-September, 2017, the fire destroyed over 300 buildings, prompted mass evacuations and burnt an estimated 192,000 hectares of forest. The notably large fire burned from Ashcroft and Cache Creek in the southwest, north to Clinton and further northwest, then over to Sheridan Lake in the northeast and south along the margin of the Deadman Valley. The region is sparsely populated and has complex terrain with limited access capability. The elevations in the area range from 278 m to 1785 m with a mean elevation of 1142 m and a standard deviation of 224.9 m. Further relating the topographic complexity of the terrain, the slopes vary from 0° to 71° with the mean slope of 8.1° and standard deviation of 6.85°. The major land cover classes include forest, grassland, clear cuts, barren land and settlements.

The Camp Fire was the deadliest and most destructive wildfire in California history. Started on November 8 near Camp Creek Road in Butte County, northern California and contained on November 25, 2018, the fire caused at least 85 civilian fatalities, 3 missing and 17 injured, and the evacuation of 52000 people. The fire also destroyed 18804 structures and burnt over 62000 hectares. The area is located in the foothills of the Sierra Nevada and the Sierra Nevada mountain range in the western United States. Compared to Elephant Hill, this region has steep complex mountainous terrain. The elevations in the area range from 49 m to 1569 m with a mean elevation of 551 m and a standard deviation of 325 m. The slopes vary from 0° to 72°. The mean slope is 14.6°, double that of the Elephant Hill, with a standard deviation at 9.91°. The major land cover types include forest, grassland, barren land, crop land and settlements.

**Figure 1:**
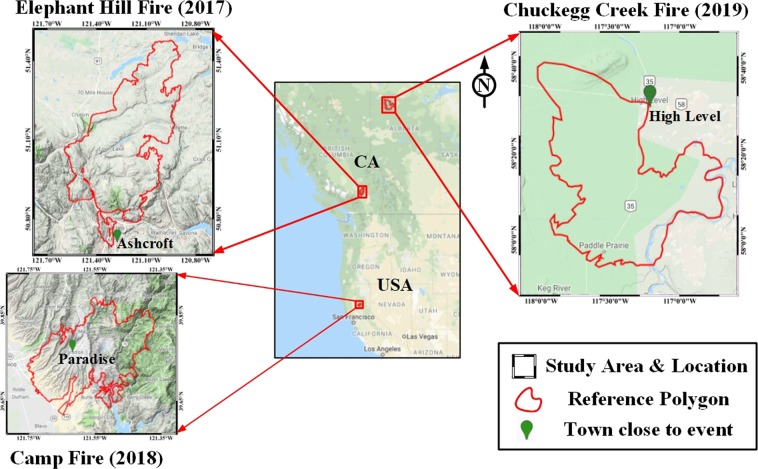
Study areas: Elephant Hill Fire (Canada, 2017), Camp fire (USA, 2018) and Chuckegg Creek Fire (Canada, 2019). The maps of the study areas were generated using ArcGIS 10.7.1 software based on the Terrain Base Map layer in Google Earth Engine (Map data ©2019 Google).

The Chuckegg Creek Fire, located west of High Level, was the largest forest fire in Alberta in 2019. Started on May 17, 2019 and contained on July 26, 2019, the fire burned an estimated 350,134 hectares of vegetation. Compared to the above two study areas, the terrain in the region is less complex and relatively flat. The elevations in the area range from 248 m to 796 m with a mean elevation of 375.5 m and a standard deviation of 55.4 m. The corresponding slopes vary from 0° to 60° with a mean slope of 2.25° and a standard deviation of 2.06°. The major land covers include forest, shrubland, grassland, wetland and barren land. The geography and topography of the three study areas are shown in Fig. 1.

### Data description

#### Sentinel-1 SAR data

The Sentinel-1 mission, the first of European Space Agency’s five Copernicus Missions, is a constellation of two polar-orbiting satellites launched on April 3, 2014 and April 25, 2016 respectively. The C-band Synthetic Aperture Radar (SAR) operates day and night in dual polarizations and acquires imagery continuously regardless of weather with a 6-day revisit at equator ^46^ (ESA, 2019). In this study, all available Sentinel-1 SAR C-band time series data in VV and HV polarizations, acquired in Interferometric Wide Swath (IW) mode, were collected for the Elephant Hill Fire, Camp Fire and Chuckegg Creek Fire sites during 2017, 2018 and 2019 respectively. Downloaded from the Google Earth Engine (GEE) Platform^47^, the Level 1 Ground Range Detected (GRD) products consist of focused SAR data that has been detected, multi-looked and projected to ground range using an Earth ellipsoid model. The SAR data were then terrain corrected converted to decibels (dB) via log scaling.

#### Reference data

**Figure 2:**
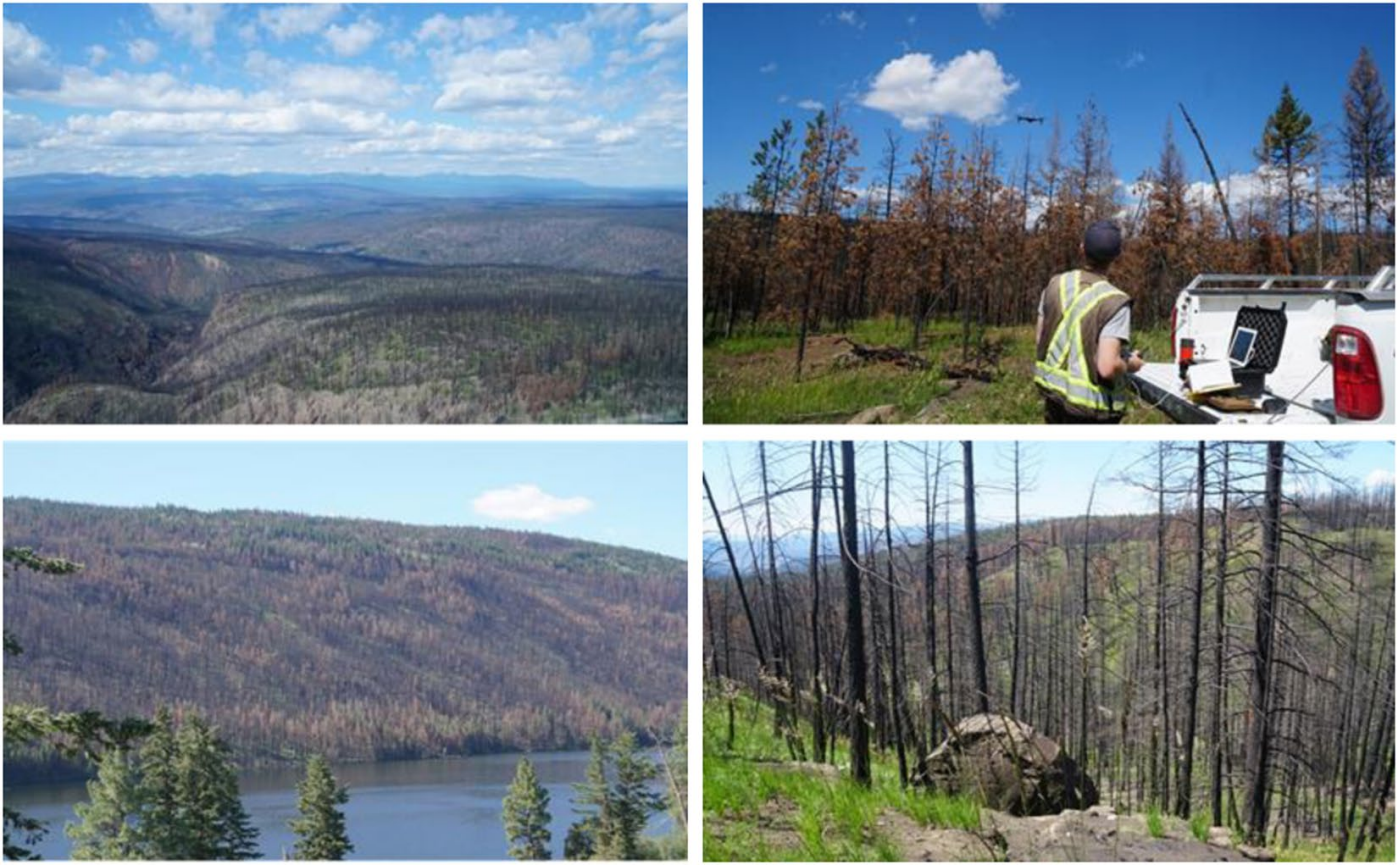
Burn severities in various terrain conditions shown in photos from fieldwork at the Elephent Hill Fire (July 18–19, 2018).

High resolution Worldview-3 post-fire imagery over the Elephant Hill Fire site was acquired on September 28, 2017 and used to visually verify the SAR-based burnt area maps. Fieldwork was also conducted in the Elephant Hill Fire area in July, 2018, one-year after the wildfire. Ground truth data representing various burn severities were collected by field inspection and with a drone. Figure 2 shows various burn severities under different terrain conditions.

To verify and validate the SAR-based mapping results, cloud-free Sentinel-2 Multispectral Instrument (MSI) imagery before, during, and after the wildfires were selected in the Elephant Hill Fire in 2017, the Camp Fire in 2018 and the Chuckegg Creek Fire in 2019. For validation, burnt areas were automatic extracted using pre-fire and post-fire Normalized Burn Ratio (NBR) and their difference (denoted as dNBR) using Eqs. (1) and (2)^48^. For each study area, the accuracy of the SAR-based final burnt area map was quantitatively assessed using 20,000 validation points (10,000 points each for burnt and unburnt areas respectively) randomly selected based on the burnt area map derived from the post-fire Sentinel-2 imagery. It should be noted that the randomly selected training and validation data sets are from the same geographical region. Therefore there is a slight chance that a few of the validation samples overlap or in close vicinity of the training samples. This may affect the overall accuracy of the mapping results. Due to lack of ground truth data during the wildfires and no Sentinel-2 or Landsat imagery to couple with each SAR acquisition during the wildfires, the SAR-based progression maps were visually compared with the burnt area maps that were derived from Sentinel-2 imagery acquired right after each acquisition date of the SAR data.

1 $${\rm{NBR}}=\frac{{\rm{NIR}}-{{\rm{SWIR}}}_{2}}{{\rm{NIR}}+{{\rm{SWIR}}}_{2}}$$

2 $${\rm{dNBR}}={{\rm{NBR}}}_{{\rm{pre}} \mbox{-} {\rm{fire}}}-{{\rm{NBR}}}_{{\rm{post}} \mbox{-} {\rm{fire}}}$$

To better understand the behavior of SAR temporal backscatter under different conditions, precipitation data over the study areas was collected. For the Elephant Hill Fire, the precipitation data used is PERSIANN-CDR, a daily precipitation estimation from remotely sensed information with artificial neural network-Climate Data Record, at a resolution of 0.25 arc degrees^49,50^. For the Camp Fire, the precipitation data is from the Climate Hazards Group InfraRed Precipitation with Station data (CHIRPS), which is a 30 year quasi-global daily rainfall dataset and it incorporates 0.05° resolution satellite imagery with *in-situ* station data^51^.

## Results and Discussion

### Sentinel-1 SAR temporal backscatter patterns of burnt and unburnt vegetation

To better understand the SAR backscatter behaviors of burnt and unburnt vegetation, several areas of interest (AOI) on SAR time series representing forest and grassland in similar vegetation (pre-fire) and topographic conditions (elevation, slope, aspect) were selected for analysis and comparison of their temporal backscatter patterns. The SAR backscatter statistics corresponding to several comparable pairs of AOIs for burnt and unburnt forest and grassland is presented in Table 1. For each AOI, the location, size and topographic information as well as their temporal means and standard deviations of SAR backscatter are listed. The reason why we use differently sized AOIs for these two fire events is that the terrain of the Camp Fire is much more complex and locally dynamic than that of the Elephant Hill Fire. Therefore, the smaller AOIs are more homogenous and representative.

**Table 1:** SAR backscatter statistics of burnt and unburnt forest and grassland.

Fire Event	Land Cover		AOI			Topography			C-VH (dB)		C-VV (dB)	
			long. (°)	lat. (°)	size (m)	elevation(m)	slope (°)	aspect (°)	mean	stdDev	mean	stdDev
Elephant Hill (ASC-64)	forest	unburnt	121.43W	50.85N	300	1120	16.80	60	−17.40	0.71	−11.37	0.49
		burnt	121.35W	50.78N		1074	16.25	71	−20.17	1.17	−13.63	0.74
	grass	unburnt	121.38W	50.69N		790	5.12	135	−24.11	1.04	−16.40	0.49
		burnt	121.33W	50.75N		748	5.36	136	−26.84	0.80	−18.60	1.08
Camp Fire (ASC-137)	forest	unburnt	121.38W	39.73N	100	769	11.64	113	−16.64	0.86	−10.75	0.65
		burnt	121.53W	39.79N		729	5.06	108	−14.53	0.89	−8.25	0.73
	grass	unburnt	121.76W	39.75N		170	5.68	163	−19.11	1.35	−10.82	0.45
		burnt	121.70W	39.69N		171	3.49	161	−19.53	1.29	−7.77	0.62

**Figure 3:**
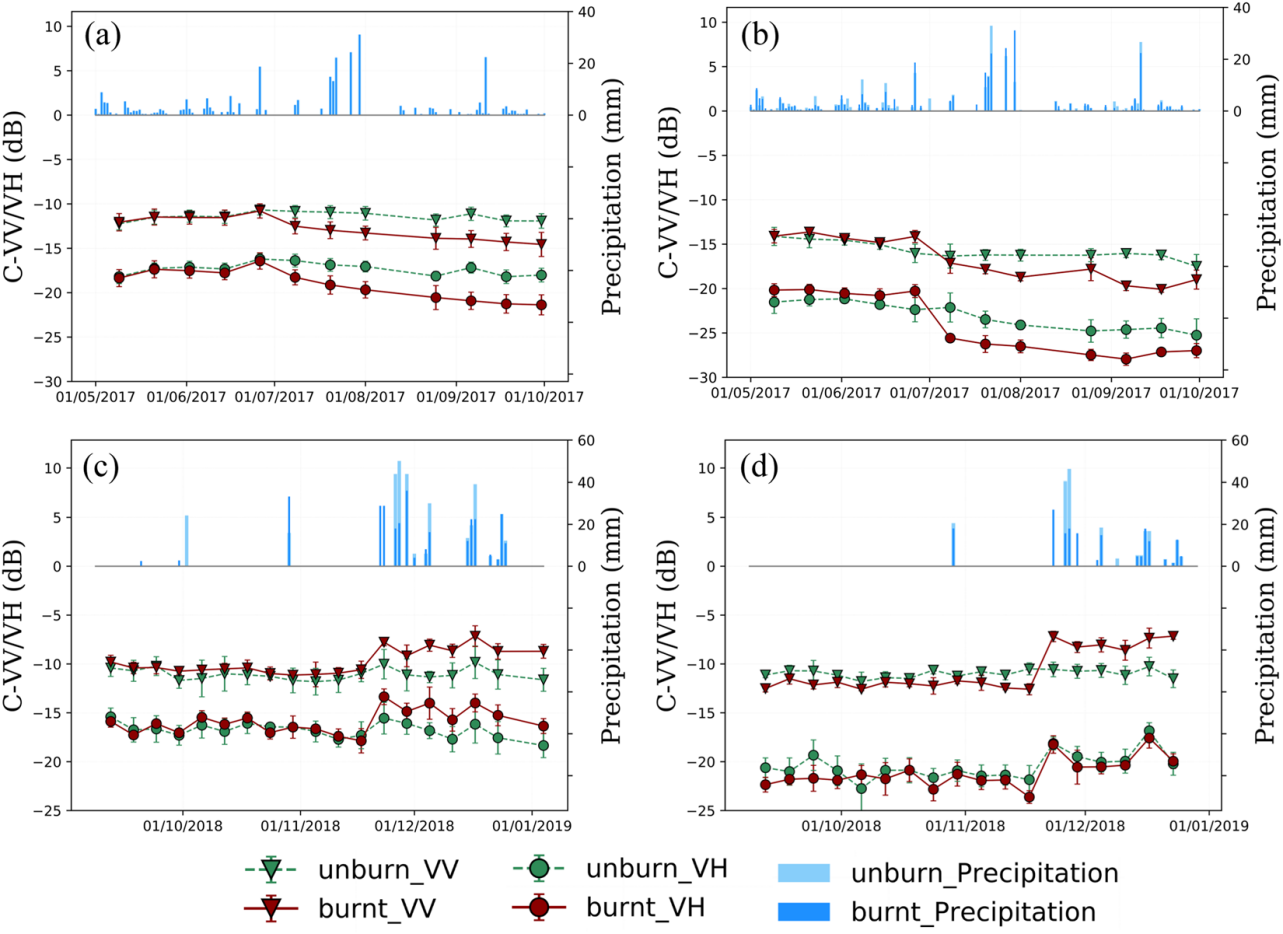
SAR backscatter varies over time in four pairs of unburnt and burnt AOIs, each of which are with similar terrain and same vegetation type. SAR backscatter behavior in two comparable. (**a**) Forest AOIs in the Elephant Hill (ASC-64). (**b**) Grass AOIs in the Elephant Hill (ASC-64). (**c**) Forest AOIs in the Camp fire (ASC-137). (**d**) Grass AOIs in the Camp fire (ASC-137).

The examples of the SAR temporal backscatter behaviors of burnt and unburnt forest and grassland, corresponding to the AOIs listed in Table 1 and shown in Fig. 3. For each pair of the AOIs, the daily precipitation histograms are also shown in this figure since vegetation wetness and soil moisture conditions could impact the SAR backscatter. Figure 3(a,b) show the SAR backscatter variations over time for forest and grassland respectively in Elephant Hill Fire site while (c) and (d) display the temporal backscatter variations in the Camp Fire site. Figure 3(a) shows that, before the wildfire event on July 6, 2017, the AOIs of burnt and unburnt forest share the same SAR backscatter patterns, indicating that the forested areas have very similar vegetation and topographic conditions before the fire. After July 6th, however, the mean backscatters for both C-VH and C-VV polarization in the burnt AOI decreased approximately 2–3.5 dBs, with a larger standard deviation while the SAR mean backscatters in the unburnt AOI remain rather stable. The temporal trends of C-HVs and C-VV backscatter are very similar even though C-VV backscatter coefficients are several dBs higher than that of C-VH. The precipitation events did not affect the backscatter for either burnt or unburnt forest as the rain events were not right before the SAR data acquisitions. Similarly, burnt AOI shares similar backscatter patterns to unburnt AOI before fire in the forest AOIs of the Camp Fire site. However, both C-VV and C-VH backscatter coefficients increased after the fire event, as shown in Fig. 3(c). The increase in backscatter of burnt forest is likely caused by rain events immediately before the SAR data acquisitions resulting in higher soil moisture contents in burnt areas.

For the grassland AOIs in the Elephant Hill Fire site, similar temporal backscatter patterns to forest are observed, as shown in Fig. 3(b). The mean backscatters of the burnt AOI decreased while that of unburnt AOI remain relatively stable. Again, the precipitation events did not affect the SAR backscatter as the rain events were not right before the SAR data acquisitions. For the grassland AOIs in the Camp Fire site, as shown in Fig. 3(d), obvious increases in C-VV backscatters after the start of the wildfire are observed due to rain events, similar to forest backscatter increase in the Camp Fire site. Several heavy rain events occurred between Nov. 21 and Nov. 28 of 2018, and VV is more sensitive to the soil moisture than VH in burnt areas. However, the C-VH backscatter patterns of unburnt and burnt grassland remain the same after the fire. The reasons for this need further investigation as C-band radar signature is usually not significantly influenced by dry grass subject to fire event according to literature. This observation further confirms the previous findings that identification of the burnt area for non-forest vegetation (i.e., grassland) is not so straightforward.

### Sentinel-1 SAR for near real-time wildfire progression monitoring

#### Elephant hill fire

**Figure 4:**
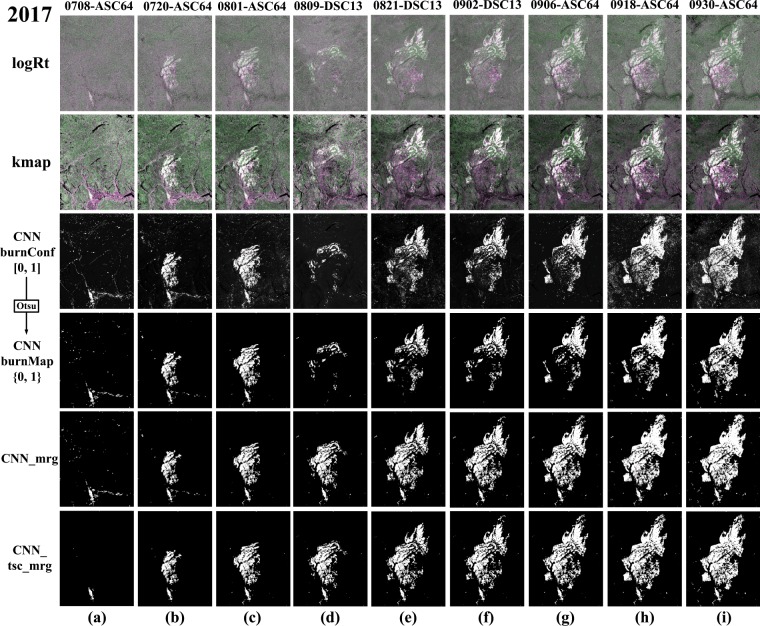
SAR-based results by three different methods on the Elephant fire. *logRt* is the false color composite with [Δ*β*_VH_, Δ*β*_VV_, Δ*β*_VH_], while the second row shows the false color composite of *kmap* with [*k*_VH_, *k*_VV_, *k*_VH_]. The third row shows the burn confidence map predicted by CNN (denoted as *CNN burnConf*), and the fourth row are the corresponding binary burn map, marked with *CNN burnMap*. *CNN*_*mrg* is the accumulated burn map before the considering date, while *CNN*_*tsc*_*mrg* are produced with time series correction, following the merging operation same as *CNN*_*mrg*.

The SAR-based wildfire progression maps of the Elephant Hill Fire are presented in Fig. 4, estimated by different methods, including the classical log-ratio (logRt)^35^, the ratio between the logRt and the corresponding historical stdDev map (kmap) and CNN-based framework. The first three rows show the estimated change maps, while the fourth row shows the binary progression maps corresponding to the burn confidence maps predicted by CNN in the third row. Row 5, labeled as *CNN*_*mrg*, presents the merged progression maps for each date, produced by accumulating all the progression maps before a date. To reduce unnecessary noises, the merging operation was not applied on the first two dates, i.e., July 8 and July 20, 2017. In the last row, *CNN*_*tsc*_*mrg* denotes that a simple time series correction (TSC) was applied on the progression maps in the same orbit before merging them. It is assumed the burnt area would not disappear in the later progression once it appeared before, TSC can be used to reduce the noisy pixels in the earlier progression maps based on the later progression maps. However, with TSC, the *CNN*_*tsc*_*mrg* is not a near real-time approach any longer because it depends the future progression maps. For any date in the Elephant Hill Fire, the TSC was implemented by multiplying it with the future progression maps in the same orbit.

To quantitatively assess SAR-based burnt area results, Sentinel-2 dNBR is segmented into a binary map of burnt and unburnt areas and used as the reference maps together field data and WorldView-3 imagery. 10,000 validation points are randomly selected from burnt and unburnt areas respectively. Table 2 presents the quantitative evaluation of SAR-based wildfire progression results for the Elephant Hill Fire. Among logRt, kmap, *CNN_mrg* and *CNN_tsc_mrg*, *CNN_mrg* achieves the highest values in Precision, Recall, OA, Kappa and F_1_, *CNN_tsc_mrg* ranks second, and both of them are much higher than logRt and kmap-based results. It is worth noting that *CNN_tsc_mrg* reaches a very high value in Recall (0.9952), which implies that TSC greatly reduces the false alarm rate, compared to *CNN_mrg’s* Recall (0.9336).

**Table 2:** Quantitative Evaluation of SAR-based Results on the Elephant Hill Fire (2017).

Sat.	Bands	Method	Seg.	Precision	Recall	OA	Kappa	F_1_
S1	VH	**logRt**	Otsu	63.34%	97.12%	80.73%	0.6146	0.7667
	VV			59.42%	93.86%	77.78%	0.5553	0.7277
	VH	**kmap**		60.92%	98.53%	79.70%	0.5939	0.7480
	VV			57.98%	97.23%	78.17%	0.5633	0.7264
	VH, VV	**kmap**	>2	83.66%	94.71%	89.50%	0.7899	0.8884
	VH, VV	**CNN_mrg**	Otsu	**90**.**41%**	93.36%	**94**.**92%**	**0**.**8983**	**0**.**9467**
		**CNN_tsc_mrg**		85.89%	**99**.**52%**	92.74%	0.8548	0.9221

**Figure 5:**
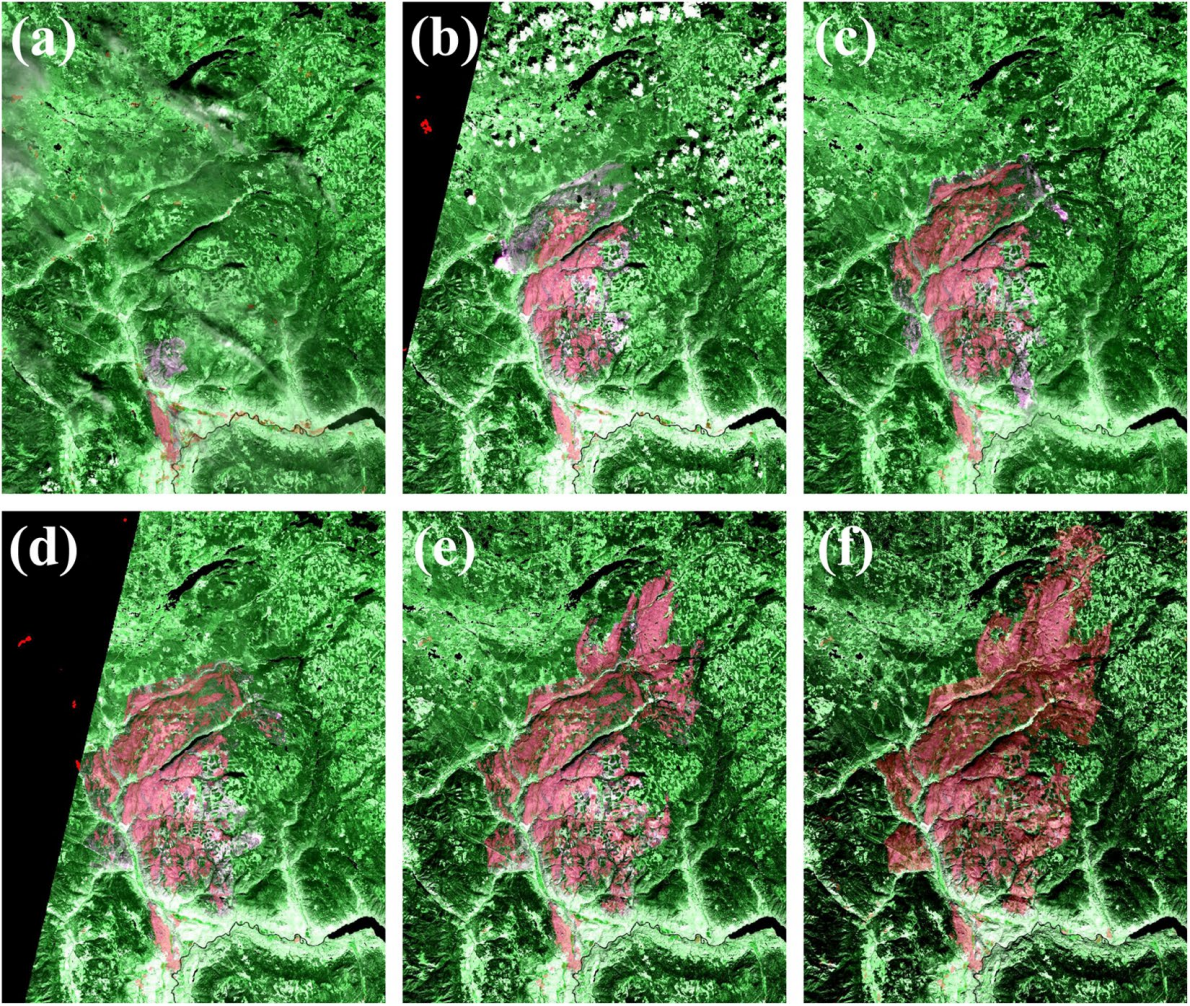
Sentinel-1 based wildfire progression maps in the Elephent Hill (*CNN*_*mrg* in transparent red) overlaid on the Sentinel-2 MSI false color composites (R = SWIR_2_, G = SWIR_1_, B = SWIR_2_). (**a**) SAR-July 8 on MSI-July 10. (**b**) SAR-July 20 on MSI-July 30. (**c**) SAR-Aug. 1 on MSI-Aug. 4. (**d**) SAR-Aug. 8 on MSI-Aug. 11. (**e**) SAR-Aug. 21 on MSI-Aug. 22. (**f**) SAR-Sept. 18 on MSI-Oct. 3. The images were generated using Google Earth Engine platform (Map data: Google, ESA).

Due to lack of field data and optical images acquired on the same date as the SAR imagery during the wildfire, the progression maps are validated visually by overlaying *CNN_mrg* on the Sentinel-2 false color composite (R = SWIR_2_, G = SWIR_1_, B = SWIR_2_). For each map in *CNN_mrg_overlay*, the Sentinel-2 image with the closest cloud-free date after the SAR acquisition. Visual observation shows that there is a high level of agreement between Sentinel-1 SAR progression map and Sentinel 2 burnt area in the full time series. The examples of the overlays are presented in Fig. 5.

#### Camp fire

**Figure 6:**
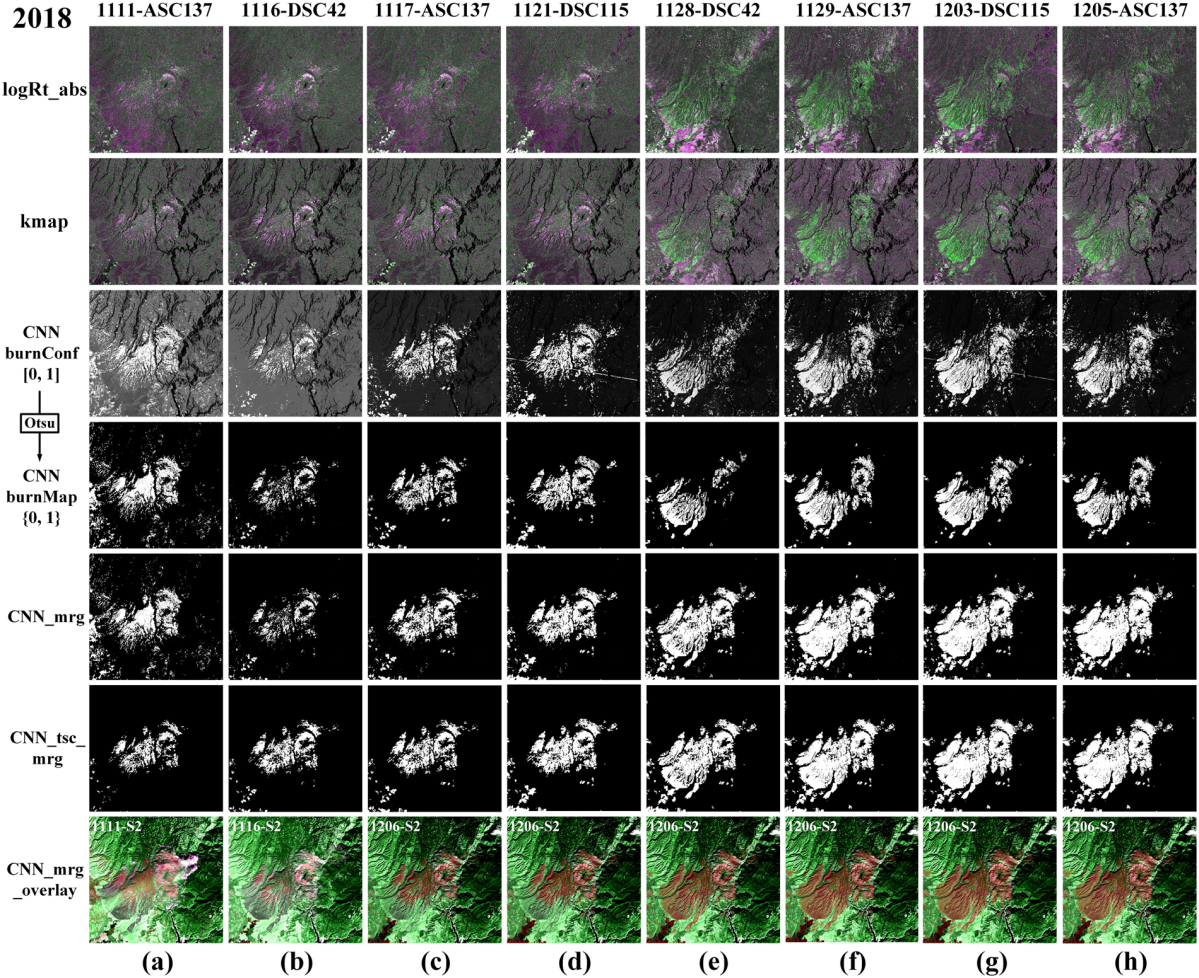
SAR-based results on the Camp fire. logRt_abs is the false color composite with [|Δ*β*_VH_|, |Δ*β*_VV_|, |Δ*β*_VH_|], while the second row shows the false color composite of kmap with [*k*_VH_, *k*_VV_, *k*_VH_]. The third row shows the burn confidence map predicted by CNN, and the fourth row lists the corresponding binary burn map, marked with CNN burnMap. *CNN_mrg* is the accumulated burn map by keeping all burn map detected CNN before the considering date, while *CNN_tsc_mrg* are produced with time series correction on the same orbit, following the merging operation same as *CNN_mrg*. The bottom row shows the overlayed maps that lay *CNN_mrg* map over the S2-based false color map with composite [R = SWIR_2_, G = SWIR_1_, B = SWIR_2_].

The SAR-based wildfire progression maps of the Camp Fire are presented in Fig. 6, estimated by different methods, including logRt with absolute operation (denoted as *logRt_abs*), kmap and CNN burnt Confidence map (*CNN burnConf*). Compared with *logRt_abs* and kmap, we can find that *CNN burnConf* highlights the burnt areas very well, that is critical for subsequent segmentation. The row marked with *CNN burnMap* shows the corresponding binary map of *CNN burnConf* maps with Otsu thresholding^52^, and *CNN_mrg* and *CNN_tsc_mrg* are produced with similar procedures for the Elephant Hill Fire. For the Camp Fire, the *logRt_abs* is exploited to detect both positive and negative backscatter changes, due to the fact that there exist both increased and decreased backscatter changes in the fire related areas. In the *logRt_abs* maps, as shown in the first row in Fig. 6, are the false color composite (R = |Δ*β*_VH_|, G = |Δ*β*_VV_|, B = |Δ*β*_VH_|).

In the first four stages, most of the burnt areas are in purple, indicating that VH backscatter is more sensitive than VV to the changes caused by the wildfire event, while the white pixels indicate that VH and VV show similar sensitivity to the changes. However, in the last four stages, the burnt area appears very different, in green instead of purple. The green pixels show that VV backscatter are more sensitive than VH to the fire-induced changes. This is because several heavy precipitations occurred between Nov. 21 and Nov. 28 of 2018, and VV is more sensitive to the soil moisture than VH in burnt areas. As shown in the *CNN burnConf* maps, the first two stages (a) and (b) indicate that CNN helps enhance the difference between burnt and unburnt areas, but significant over and under estimations are observed. As new SAR data comes, the CNN model is fine-tuned further, then the predicted *CNN burnConf* maps show much better contrast between burnt and unburnt pixels than the first two stages. With Gaussian filtering followed by Otsu thresholding^52^, *CNN burnConf* maps in range [0, 1] are binarized into *CNN burnMap*. As expected, the first stage (a) looks rather noisy and the later stages detect most of the burnt areas with less noise. *CNN_mrg* combines all detected burnt areas on different orbits before current dates, except for the first date, Nov. 11, 2017, which is too noisy. *CNN_tsc_mrg* provides non near real-time results with less false alarm pixels by applying time series correction on the same orbit. By overlaying Senintel-1 SAR-based progression results (in transparent red) and Sentinel-2 SWIR composite, the bottom row demonstrates that there is a certain degree of agreement between Senintel-1 SAR-based progression maps and Senintel-2 fire scars. Compared to the visual observations in the Elephant Hill Fire, the agreement on the Camp Fire is not as good.

Quantitative evaluations of SAR-based wildfire progression results of the Camp Fire are presented in Table 3. With kmap, C-VV achieves a much higher value than C-VH in OA, Kappa and F_1_ score, and combining C-VV and C-VH can reach a higher accuracy than VV or VH alone. By combining VH and VV, *CNN_mrg* achieves an overall accuracy of 83.58% (Kappa: 0.6716, F_1_: 0.8139), and *CNN_tsc_mrg* reaches a higher Recall value than *CNN_mrg*, i.e., a lower false alarm rate.

**Table 3:** Quantitative Evaluation of SAR-based Results on the Camp Fire (2018).

Sat.	Data	Method	Seg.	Precision	Recall	OA	Kappa	F_1_
S1	VH	**kmap**	>2	21.82%	65.13%	55.07%	0.1014	0.3269
	VV			45.21%	80.19%	67.02%	0.3404	0.5782
	VH, VV	**kmap**	>2	51.17%	82.11%	70.01%	0.4002	0.6305
	VH, VV	**CNN_mrg**	Otsu	**71**.**82%**	93.91%	**83**.**58%**	**0**.**6716**	**0**.**8139**
		**CNN_tsc_mrg**		70.60%	**95**.**07%**	83.47%	0.6694	0.8103

#### Chuckegg creek fire

**Figure 7:**
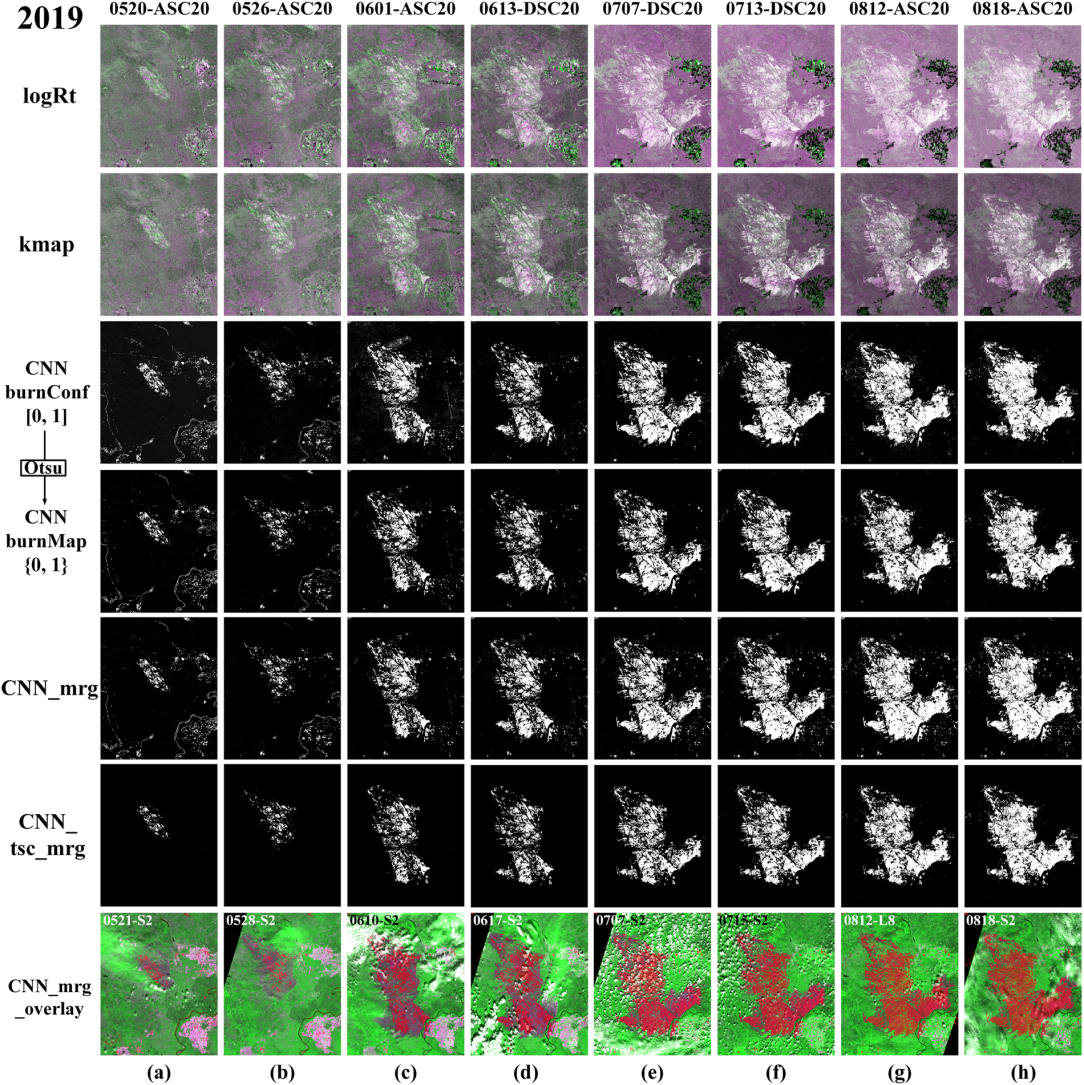
SAR-based results on the Chuckegg Creek Fire (2019). logRt is the false color composite with [Δ*β*_VH_, Δ*β*_VV_, Δ*β*_VH_], while the second row shows the false color composite of kmap with [*k*_VH_, *k*_VV_, *k*_VH_]. The third row shows the burn confidence map predicted by CNN, and the fourth row lists the corresponding binary burn map, marked with CNN burnMap. *CNN_mrg* is the accumulated burn map by keeping all burn map detected CNN before the considering date, while *CNN_tsc_mrg* are produced with time series correction on the same orbit, following the merging operation same as *CNN_mrg*. The bottom row shows the overlayed maps that lay *CNN_mrg* map over the S2-based false color map with [R = SWIR_2_, G = SWIR_1_, B = SWIR_2_].

Figure 7 presents the SAR-based wildfire progression maps of the Chunkegg Creek Fire, estimated by logRt, kmap and the proposed CNN-based deep learning framework. Different from the Elephant Hill Fire and the Camp Fire, Sentinel-1 SAR acquired images every six days in the same orbit (ASC20) over the Chuckegg Creek Fire, a higher imaging frequency. The logRt-based progression maps showed that the VV and VH backscattering have similar sensitivity to changes caused by fire, thus the burnt areas appear white. However, they have very different responses to changes caused by agricultural activities. While VH increases slightly, VV decreases significantly over the agricultural areas (in green). Similar to a wildfire event, the agricultural activities may cause a significant decrease in VV backscattering, which would result in false alarms. Owning to the fact that the agricultural fields often have a high standard deviation in the historical time series, kmap can suppress the agricultural activities-related changes better than log ratio, as shown in the second row. Trained with samples from the binarized kmap, the CNN-based framework can highlight the burnt areas and suppress false alarms due to agricultural activities, as shown in CNN_burnConf and CNN_burnMap. CNN_mrg and CNN_tsc_mrg show the merged results of the burnt areas without or with TSC respectively. The bottom row shows the visual comparison between optical images and SAR-based results, which indicate that SAR data has the potential to detect most of the burnt areas, but some low burn severity areas without structural changes may be missed.

Table 4 summarizes the quantitative analysis of the SAR-based wildfire progression mapping results, and these statistics are based on 10,000 samples randomly selected from burnt areas and unburnt areas respectively. With kmap, both VH and VV reach a very high Recall value but a low Precision value, indicating that both of them have a very low false negative rate and high false positive rate. By combining VH and VV together, kmap achieves a much higher precision without a significant decrease in Recall, resulting in the increase in OA (73.05%), Kappa (0.4609) and F1 (0.6339). By applying the proposed CNN-based framework using both VH and VV data, CNN_mrg can achieve a significant improvement in Precision with a minor decrease (0.3%) in Recall, leading to 88.09% in OA, 0.7618 in Kappa and 0.8666 in F1 score. By exploiting TSC, CNN_tsc_mrg can reduce the noisy pixels very well, but the accuracy decreases slightly.

**Table 4:** Quantitative Evaluation of SAR-based Results on the Chuckegg Creek Fire (2019).

Sat.	Data	Method	Seg.	Precision	Recall	OA	Kappa	F_1_
S1	VH	**kmap**	>2	29.34%	99.69%	64.63%	0.2925	0.4534
	VV			21.38%	99.21%	60.61%	0.2121	0.3518
	VH, VV	**kmap**	>2	46.67%	98.77%	73.05%	0.4609	0.6339
	VH, VV	**CNN_mrg**	Otsu	**77**.**38%**	98.47%	**88**.**09%**	**0**.**7618**	**0**.**8666**
		**CNN_tsc_mrg**		67.09%	**99**.**94%**	83.53%	0.6705	0.8028

## Conclusions

In this paper, we evaluated Senitnel-1 SAR time series for near real-time wildfire progression monitoring using a novel and fully automatic deep learning framework based on CNN. The analysis of SAR temporal backscatter profiles showed that significant differences between burnt forest and grassland can be observed in both the Elephant Hill Fire and the Camp Fire sites (except C-VH over the Camp Fire site). The CNN-based deep learning framework performed much better than log-ratio based kmap in detecting burnt areas, achieving a significant improvement in Kappa over these three study areas: (0.11 for the Elephant Hill Fire, 0.27 for the Camp Fire and 0.30 for the Chuckegg Creek Fire, respectively). By fine-tuning with local data, we demonstrate the proposed CNN framework is effective in monitoring the progressions of three large wildfires in different geographic regions in various topographic conditions. Additional studies are planned to further demonstrate the transferability of the CNN framework to other wildfire events via pixel-wise network forward propagation. By exploiting all the available SAR data acquired before the wildfire event to characterize the area in terms of backscatter variations due to different environmental conditions, the time series based anomaly detection method is effective in producing coarse burnt area maps that are essential for automatic training of the CNN framework. This research is the first attempt on wildfire progression monitoring using SAR time series and deep learning in challenging topographic conditions. The findings demonstrates that, using a fully automatic deep learning framework, spaceborne SAR data can play a significant role for real-time wildfire progression monitoring when the data becomes available at daily and hourly intervals with the launches of RADARSAT Constellation Missions and SAR CubeSat constellations.

## Methodology

**Figure 8:**
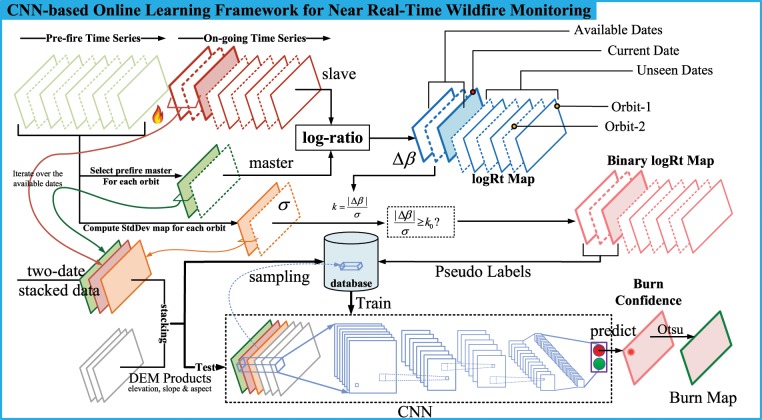
CNN-based Online Learning Framework for Near Real-Time Wildfire Monitoring.

The main goal of the methodology is to develop a novel and fully automatic procedure based on a deep learning framework that utilizes every new Sentinel-1 SAR image acquired during the wildfire event to monitor the fire progression in near real-time. When a wildfire occurs, pre-fire SAR dense time series of the study area are collected from the archive and new SAR images are acquired in near real-time during the wildfire event. In particular, the proposed method has two innovative aspects, one is to exploit all available SAR data acquired before the wildfire event to characterize the area in terms of SAR backscatter variations due to different environmental conditions (e.g., seasonal effect, different land cover, weather conditions, etc.) while the other is to automatically train an implicit deep learning framework to estimate the changes in the SAR images acquired during the wildfire. The methodology includes four major processing steps. First, log-ratio of the pre-fire and post-fire SAR images is performed to detect changes caused by wildfire. Then the coarse binary map of burnt and unburnt areas is generated using a time series based anomaly detection technique. Using training samples automatically generated from the coarse binary change map, the CNN is trained and fitted to refine the burnt area detection and to generate the burnt confidence maps. The last step is to binarize the confidence maps using the Otsu automatic thresholding approach and to combine the individual wildfire progression maps progressively to improve their reliability and consistency. The overview of the methodology is presented in Fig. 8. In the following sub-sections, a full description of the different steps is reported.

### Log-ratio based change measurement

To detect changes caused by the wildfire. comparison of pre- and post-fire SAR images is performed. For each new SAR image acquired after the start of the wildfire, a pre-fire image is selected as a master image for each available ascending (ASC) or descending (DSC) orbit. By applying log-ratio operator on the master (pre-fire) and slave (after the start of the fire) image, a change map can be derived for C-VV and C-VH respectively, and the corresponding log-ratio time series can be established. The optimal master image is selected taking into account both minimizing the seasonal effects and avoiding master images acquired after heavy rain events. Log-Ratio based change measurement is defined accordingly with the following formula:

3 $$\Delta {\beta }_{r}=10\,{\log }_{10}\left(\frac{{\beta }_{r}^{{\rm{m}}}}{{\beta }_{r}^{{\rm{s}}}}\right)=10\,{\log }_{10}({\beta }_{r}^{{\rm{m}}})-10\,{\log }_{10}({\beta }_{r}^{{\rm{s}}})$$

where *r* ∈ {ASC-*μ*, DSC-*ν*} denote the corresponding orbit direction (ASC or DSC) and relative start orbit number (*μ* or *ν*), and *β*_*r*_ is the radar backscattering value, *m* and *s* represent pre-fire (master) and post-fire (slave) image, respectively. By applying log-ratio on the master and slave images, the change map Δ*β*_*r*_ can be derived, which estimates the difference degree between master and slave images. The change map Δ*β*_*r*_ are subsequently binarized using the StdDev map as a reference estimation of the regular oscillation of the SAR backscatter. Hereafter, Δ*β*_*r*_ and *σ*_*r*_ are rewritten as Δ*β* and *σ* for convenient and compact mathematical notation.

### Time series based anomaly detection

Based on the pre-fire SAR time series, the corresponding mean and standard deviation (StdDev) are computed with respect to every pixel, forming a mean map and a StdDev map. Over the same study area, the StdDev maps *σ* are computed based on the historical SAR time series for ASC and DSC orbit respectively, including both VH and VV polarizations. The StdDev maps estimate the normal variance of SAR backscatter with seasonal changes over time, which would provide a pixel-wise reference level for different land cover types in the study area.

4 $$\begin{array}{l}\sigma (i,j)=\sqrt{\frac{{\sum }_{t=1}^{N}{({\beta }^{t}(i,j)-\bar{\beta }(i,j))}^{2}}{N-1}}\end{array}$$

where $\bar{\beta }$ represents the mean image over the available SAR time series on the same orbit, and the length is denoted as *N*, i.e., the total number of available images acquired on the same orbit.

5 $$\begin{array}{l}{I}_{k}(i,j)=\frac{|\Delta \beta (i,j)|}{\sigma (i,j)}\end{array}$$

In order to detect the abnormal variance caused by wildfire events, a k-Map can be computed by dividing *σ* from |Δ*β*(*i*, *j*)|, as shown in Eq. 5. The k-map *I*_*k*_ estimates the times that |Δ*β*(*i*, *j*)| is larger than the corresponding *σ* for each pixel in the study area: a higher value in the k-Map means it is abnormal variation corresponding to a higher probability of changes.

6 $$I(i,j)=\left\{\begin{array}{cc}1, & {I}_{k}(i,j)\ge {k}_{0}\\ 0, & {I}_{k}(i,j) < {k}_{0}\end{array}\right.$$

As illustrated in Eq. (6), the *I*_*k*_ map can be transformed into a binary map *I* with a threshold *k*_0_, which means the pixels will be considered as abnormal ones (i.e., burnt pixels) if they are larger than the *k*_0_ value in the estimated log-ratio map, otherwise, they will be taken as the normal ones, i.e., unburnt pixels. In practice, *k*_0_ = 2 is a good trade-off between detecting abnormal changes and suppressing noise. The produced binary maps are the main input in the next CNN refinement step as reference data to automatically select the training samples.

### Deep learning-based burnt area refinement

As shown in Fig. 8, we use the binary logRt map time series to select burnt and unburnt samples for training a CNN model to detect burnt area automatically. By iterating over the available dates, each image is stacked with the corresponding master image and StdDev map in the same orbit, and the DEM products such as elevation, slope and aspects can also be stacked on them. The same number of training samples are randomly chosen from the burnt areas and unburnt areas identified by the log-ratio algorithm with StdDev binarization, and these training samples are stored into a database, used to train a CNN model to further refine the burnt areas. In the testing phase, when the image stack is fed into this CNN model, the corresponding burnt area mapping will be generated automatically.

The CNN framework is designed to produce a confidence map characterized by a bi-modal distribution of burn and un-burnt pixels. Let **F**(*θ*) denotes the learnable deep network for detecting burnt areas, we can derive the output ${{\bf{O}}}_{i}^{l}$ of the first *l* layers by forward passing patch **P**_*i*_ in network **F**(*θ*) (short for **F**^*L*^(*θ*), where *L* is the total number of network layers), and the forward passing is denoted as ⊗ in Eq. (7).

7 $${{\bf{O}}}_{i}^{l}={{\bf{P}}}_{i}\otimes {{\bf{F}}}^{l}(\theta )$$

where **F**^*l*^(*θ*) denotes the first *l* layers of **F**(*θ*), and *θ* collects the weights and bias of **F**^*l*^(*θ*), *l* = 0, 1, 2, …, *L*. Specially, the input layer is taken as layer 0, and ${{\bf{O}}}_{i}^{0}={{\bf{P}}}_{i}$. So, the relationship between ${{\bf{O}}}_{i}^{l}$ and ${{\bf{O}}}_{i}^{l-1}$ can be formulated as:

8 $${{\bf{O}}}_{i}^{l}=\delta ({W}^{l}\,\ast \,{{\bf{O}}}_{i}^{l-1}+{b}^{l})$$

where *W*^*l*^ and *b*^*l*^ represent weights and bias of *l*-th layer in **F**(*θ*), respectively, and *δ* is the ReLU activation function^38^. Table 5 lists the CNN architecture used, it has 18 layers of neural networks, and each convolutional layer is followed by a ReLU activation (sigmoid for the last layer). The *CNN burnConf* map is derived by applying the sigmoid activation on the output of the last layer, since burnt area detection is actually a binary classification problem, and the sigmoid activation is a good choice to scale the predicted confidence into the range [0, 1].

**Table 5:** CNN Architecture (18 layers, learning rate: 0.0001, optimizer: Adam^53^, epoch: 20).

Layer No.	Operation	Output	
		Train	Test
0 (input)	—	7 × 55 × 55	7 × (w + 54) × (h + 54)
1	20@3 × 3Conv + ReLU	20 × 53 × 53	20 × (w + 52) × (h + 52)
2–8	20@7 × 7Conv + ReLU	20 × 17 × 17	20 × (w + 16) × (h + 16)
9–17	20@3 × 3Conv + ReLU	20 × 1 × 1	20 × w × h
18	2@1 × 1Conv + Sigmoid	2 × 1 × 1	2 × w × h

With randomly sampled data (**P**_*i*_, **y**_*i*_), a CNN-based non-linear change indicator **F**(*θ*) can be learnt for highlighting the burnt areas based on the SAR data. The predicted burn confidence vector ${\hat{{\bf{y}}}}_{i}\in {R}^{2\times 1}$ can be derived by squeezing ${{\bf{O}}}_{i}^{L}={{\bf{P}}}_{i}\otimes {\bf{F}}(\theta )\in {R}^{2\times 1\times 1\times 1}$. Therefore, the loss function can be formulated as:

9 $$\ell (\theta )=\frac{1}{n}\mathop{\sum }\limits_{i=1}^{n}\,{\Vert {\hat{{\bf{y}}}}_{i}-{{\bf{y}}}_{i}\Vert }_{2}^{2}+\lambda {\Vert \theta \Vert }_{2}^{2}$$

where *n* is the number of training samples, *θ* is the learn-able network parameter over all layers, including weights and bias, and *λ* controls the weight decay rate. In our experiment, we set *λ* = 0.001. Once trained, the corresponding burn confidence map can be obtained, which can be used to update the binary logRt time series for next training. Like this, the Pseudo label updating can contribute to providing more reliable ones, but it is not necessary. Moreover, Digital Elevation Model (DEM) products can be integrated to take topography into consideration as additional input layers.

### CNN BurnConf maps: binarization and time series merging

**Figure 9:**
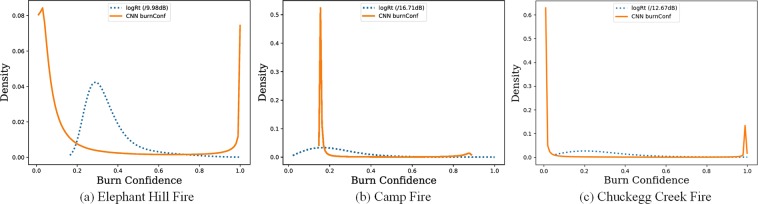
Comparison among the distribution of *CNN burnConf* and *logRT* maps on the final stage.

The outputs of the CNN refinement are *CNN burnConf* maps, where the pixel values are ranging from 0 to 1 and they are proportional to the probability that each pixel represent a burnt area (0: unburnt, 1: burnt). The main advantage of using the proposed CNN framework is that the differences in term of backscatter variation between burnt and unburnt pixel are represented by a clear bi-modal distribution (see Fig. 9) with respect to unimodal distribution of the log-ratio based results (scaled to [0, 1] by dividing the maximum). Consequently, the *CNN burnConf* maps are easy to be binarized using an Otsu automatic thresholding technique^52^.

To produce more reliable and consistent fire progression maps, the binary wildfire progression maps at different stages are combined using two different methods. The first method is for near-real time wildfire progression monitoring and it simply combines the new wildfire progression map with the previous ones to generate the latest burnt area map. This method does not use the later burnt map to improve the results of the previous ones. This method has been investigated to highlight the potential of the SAR-based CNN framework for near real-time wildfire monitoring. The second method is a post-processing step that uses all the generated binary maps to update the fire progression maps exploiting the available multitemporal information. The method reduces the noise using a gaussian temporal filtering of the produced burnt map time series and it can be used to obtain a more reliable delineation of the fire progression for post-fire analysis (i.e. calibration of fire progression models).

## Data Availability

The datasets generated during and/or analysed during the current study are available from the corresponding author on reasonable request.
